# The Cambridge Behavioural Inventory revised

**DOI:** 10.1590/S1980-57642009DN20200005

**Published:** 2008

**Authors:** Helen J. Wear, Catherine J. Wedderburn, Eneida Mioshi, Caroline H. Williams-Gray, Sarah L. Mason, Roger A. Barker, John R. Hodges

**Affiliations:** 1 BA - MRCP PhD, Cambridge Centre for Brain Repair, Department of Clinical Neurosciences, University of Cambridge, Cambridge, UK.; 2 FedMedSci, MSc OTR - Prince of Wales Medical Research Institute, Sydney, Australia.; 3 MRCP PhD, Department of Neurology, Addenbrooke’s Hospital, Cambridge, CB2 2QQ. John Hodges and Eneida Mioshi were based at the Department of Clinical Neurosciences at the time of the study.

**Keywords:** Cambridge Behavioural Inventory, neuropsychiatric symptoms, differential diagnosis of dementia, frontotemporal dementia, Alzheimer’s disease, Huntington’s disease, Parkinson’s disease

## Abstract

Neurobehavioural and psychiatric symptoms are common in a range of neurodegenerative disorders with distinct profiles which are helpful in the diagnosis and monitoring of these disorders. The Cambridge Behavioural Inventory (CBI) has been shown to distinguish frontotemporal dementia (FTD), Alzheimer’s disease (AD), Huntington’s disease (HD) and Parkinson’s disease (PD), but it is lengthy.

**Objective:**

To develop a shorter version of the 81 item CBI.

**Methods:**

CBI data from 450 participants with behavioural variant frontotemporal dementia (bv-FTD) (64), AD (96), PD (215) and HD (75) were analysed using Principal Components Analysis and measures of internal consistency (Cronbach alpha).

**Results:**

A reduced 45-item questionnaire was developed. The instrument identified distinct behavioural profiles and performed as well as the original version.

**Conclusions:**

A shorter (45 item) version of the CBI is capable of differentiating bv-FTD and AD from PD and HD. It may be useful in delineating the type and extent of problems in these disorders as well as monitoring therapeutic interventions.

## Abstract (pt)

Sintomas psiquiátricos e de comportamento são comuns em várias doenças neurodegenerativas, com perfis distintos que são úteis no diagnóstico e no acompanhamento destas doenças. O Inventário Comportamental de Cambridge (CBI) tem mostrado como distinguir entre demência frontotemporal (DFT), doença de Alzheimer (DA), doença de Huntington (DH) e doença de Parkinson (DP), mas é um instrumento longo.

**Objetivo:**

Desenvolver uma versão curta do CBI de 81 itens.

**Métodos:**

Dados de 450 participantes com a variante comportamental (VC) da DFT (64), DA (96), DP (215) e DH (75) foram analisados usando análise de componentes principais (PCA) e medidas de consistência interna (Cronbach alpha).

**Resultados:**

Uma versão reduzida de 45 itens do questionário foi desenvolvida. O instrumento identificou perfis distintos de comportamento e seu desempenho foi tão bom quanto a versão original.

**Conclusões:**

Uma versão curta (45 itens) do CBI é capaz de diferenciar VC-DFT e DA de DP e DH, e pode ser útil em identificar o tipo e extensão de problemas nestas doenças e também no monitoramento de intervenções terapêuticas.

Behavioural changes are increasingly described in progressive neurodegenerative diseases. In Alzheimer’s disease (AD), common symptoms include delusions, irritability, agitation, anxiety and depression.^1-3^ In frontotemporal dementia (FTD), changes in personality and social conduct are core to the diagnostic criteria, and include mental rigidity, stereotypical behaviour, disinhibition, apathy, and alteration in eating habits.^4,5^ Parkinson’s disease (PD) is also associated with a range of neuropsychiatric symptoms such as depression, anxiety, apathy, psychosis and visual hallucinations,^6^ the last of which is predominant in dementia with Lewy Bodies.^7^ Similarly, Huntington’s disease (HD) may present with depression, irritability and aggressive behaviour^8^ in addition to the well- known motor symptoms. Identifying and quantifying behavioural changes is not only important for differential diagnosis, but also for evaluating disease progression and for monitoring of treatment changes.

Several scales are available to detect behavioural change, perhaps the best known of which is the Neuropsychiatric Inventory (NPI).^9^ The NPI is a carer-based interview designed to detect a range of neuropsychiatric features, however, it requires training to administer and score and can be timeconsuming, which limits its applicability in clinical practice.

The Cambridge Behavioural Inventory (CBI) is an informant-based questionnaire comprising 81-items aimed at assessing behavioural changes across a range of neurodegenerative disorders.^10^ It was designed to capture cognitive, behavioural and affective symptoms as well as activities of daily living (ADL) and evaluates 13 functional/behavioural domains: memory, orientation and attention, everyday skills, self care, mood, challenging behaviour, disinhibition, eating habits, sleep, stereotypic and motor behaviour, motivation, insight and awareness. The CBI rates the frequency of any particular behaviour on a scale of 0-4. A score of zero denotes no impairment, a score of 1 an occasional occurrence (a few times per month), 2 a repeated occurrence (a few times per week), 3 a daily occurrence, and 4 constant occurrence; the latter two scores signifying a severe behavioural deficit.

The CBI was developed to detect the unique constellation of symptoms seen in FTD and was based on an earlier questionnaire.^4^ It has since been shown to distinguish between distinct profiles of behaviour seen in neurodegenerative disorders, notably FTD, AD, PD, and HD, and has been validated against the NPI.^10,11^ However, the CBI is relatively long and it is likely that a number of the questions are redundant or rarely endorsed. The aim of this study was to see if we could derive a shorter, more user-friendly version of the CBI that would still maintain its ability to detect behavioural dysfunction as well as its ability to identify disease specific patterns of behaviour.

## Methods

### Participants

Retrospective data from 450 patients seen over 10 years in Cambridge, UK were included. Neurological diagnoses included Parkinson’s disease (PD, n=215), Huntington’s disease (HD, n=75), Alzheimer’s disease (AD, n=96) and behavioural variant frontotemporal dementia (bv-FTD, n=64). All patients met internationally recognised diagnostic criteria;^12-14^ HD patients had genetically confirmed disease. Patients with uncertain diagnoses were excluded. Detailed methods of diagnostic criteria and data collection have been described elsewhere.^10^ All patients had undergone the Mini-Mental State Examination (MMSE)^15^ at the time of completing the CBI. The study was approved by the Cambridge Local Research Ethics Committee.

### Data analyses

#### Reduction of the CBI

Several steps were taken in the process of revising the questionnaire:

1. A principal components analysis (PCA) using varimax rotation was performed on all the data from the 81-item questionnaire. This method is widely used to reduce the dimensionality of a data set.^16^ In a factor analysis, items that correlate highly with each other group together in clusters (factors) that are considered to reflect underlying constructs. Factors accounting for 3% or less of variance were discarded and factors with Eigenvalues >1 were retained. Meaningful factor loadings were considered to be r>0.50and any item that did not load sufficiently onto a factor was removed.
2. Compliance for each item in the CBI was examined by calculating the percentage of carers that gave an answer of 0-4 rather than N/A (not applicable) or leaving a blank. Very poor compliance for certain items would result in a significant bias in the mean score for the subsection, and so questions with a compliance of less than 80% were removed.
3. The percentage of patients with a score of zero was then calculated for each question. >90% zeros indicated that the question was relatively insensitive in all diseases, hence that question was excluded.
4. In consultation with the designer of the original instrument (JRH) and with evidence from the literature, further inspection for redundancy was undertaken. Items that clustered together on the same PCA factor and that were not deemed to be clinically relevant were removed.

#### Validity and utility of the Revised Cambridge Behavioural Inventory (CBI-R)

The retained items for the revised questionnaire were again subjected to a PCA to confirm that the underlying factor structure remained the same. Preliminary validity and utility analyses were also performed. This involved comparing the internal consistency of the original questionnaire subsections,^10^ assessed using data from 450 patients, with the internal consistency of the revised questionnaire. Adequate internal consistency infers that all the items on the questionnaire that make up a composite score reflect the same underlying construct. Internal consistency was calculated using Cronbach’s α, where values range from 0-1, with higher scores reflecting higher internal consistency.^17^ *T* tests were performed to compare the subsections from the original CBI with the revised version. The diagnostic value of the revised questionnaire was also assessed by investigating the distribution of behavioural deficits in the four neurodegenerative diseases. Behavioural profiles were created using data from the original 450 patients, but excluding the questions that had been removed. These were then compared with behavioural profiles drawn up using the original questionnaire.^10^

## Results

### Patient characteristics

Sociodemographic, cognitive and behavioural characteristics of the 450 patients are shown in Table 1. Age and proportion of male patients differed significantly between groups. HD patients were on average younger than any other group, whereas bv-FTD patients presented with the highest discrepancy in the proportion of male to female patients. The bv-FTD group had the highest endorsements on the CBI. There was also a significant difference on MMSE scores, the AD group being the most impaired.

**Table 1:** Demographic, cognitive and behavioural data on the patients involved in the study. Range in brackets.

Diagnosis	Mean age (years)	Gender Male/Female	Mean CBI score	Mean MMSE score
PD (n=215)	68.3 (37.1-90.1)	137 / 78	35 (1-140)	27.2 (8-30)
HD (n=75)	52.4 (21-79.8)	33 / 42	62 (2-189)	25.1 (12-30)
AD (n=96)	67.0 (47- 90)	53 / 43	78 (6-230)	17.9 (4-27)
Bv-FTD (n=64)	62.0 (43-81)	47 / 17	114 (10-227)	21.2 (2-30)
ANOVA				
F	45.49	7.65	61.14	9.86
p	0.000	0.000	0.000	0.000

AD: Alzheimer's disease; Bv-FTD: behavioural variant frontotemporal dementia; CBI: Cambridge Behavioural Inventory; HD: Huntington's disease; MMSE: mini-mental state examination; PD: Parkinson's disease.

### Reduction of the CBI

The initial PCA conducted on the 81-item questionnaire data (n=450) produced 15 factors with eigenvalues >1, which accounted for 67.5% of the variance. Seven factors were discarded as they each accounted for less than 3% of the total variance. Items that did not load sufficiently onto one of the eight retained factors were removed, [thereby retaining all significant factors with loadings of r>0.50^16^]. This method resulted in the deletion of 20 questions. In two cases, loadings were just below the cut off (r>0.47), but the questions were considered clinically relevant and so were retained.^4^

The majority of questions had high compliance rates. Three questions from the ‘everyday skills’ subsection had a compliance of <80% and these were removed. Analysis of the percentage of zeros revealed 6 questions scored as zero by >90% of patients and these were deleted. Redundant items which were clustered were also discarded. The only full section deleted from the original questionnaire was ‘insight and awareness’, because none of the questions loaded sufficiently onto one of the eight retained factors. Additionally, the scoring system for this section was confusing as it did not concur with the rest of the questionnaire.

Based on clinical experience and evidence in the literature,^4,5^ we retained questions 54-57 in the ‘eating habits’ section of the original CBI despite these questions not loading onto a factor. Similarly, we also retained the two questions in the ‘sleep’ section, as sleep disorders are increasingly being recognised as a symptom in a range of neurodegenerative diseases.^6^

### Revised Cambridge Behavioural Inventory (CBI-R)

The retained 45 items were subjected again to PCA to confirm the factor structure. The repeat PCA yielded nine factors with Eigenvalues >1, each accounting for over 3% of the variance (Table 2). The item loadings were similar to that of the initial PCA. All questions loaded onto at least one factor, including those representing ‘sleep’ and ‘eating habits’, supporting the decision to retain them in the revised questionnaire.

**Table 2:** Principal components analysis on the revised questionnaire (total variance explained); internal consistency of the Cambridge Behavioural Inventory revised, as measured by Cronbach's alpha.

Factor	Total eigenvalues	% of variance	Cumulative %	Cronbach alpha new groupings
1 = Memory and orientation and attention	6.7	14.8	14.8	0.93
2 = Challenging behaviour and disinhibition	5.1	11.2	26.1	0.87
3 = Everyday skills and self-care	4.3	9.5	35.5	0.89
4 = Motivation	3.9	8.7	44.2	0.91
5 = Mood	2.5	5.5	49.7	0.82
6 = Eating behaviour	2.5	5.5	55.2	0.76
7 = Abnormal beliefs	2.1	4.7	59.8	0.77
8 = Stereotypic and motor behaviours	1.8	3.9	63.8	0.69
9 = Sleep	1.67	3.8	67.5	0.58

A Cronbach’s alpha test performed on the revised questionnaire indicated a high degree of internal consistency (Table 2), comparable to the original CBI. Only the sleep subsection had a Cronbach alpha below the recommended level (α<0.7) for group comparisons in research situations.^17^

The PCA factor structure was used to regroup the retained questions into new subsections for the revised questionnaire, named on the basis of the predominant theme of the questions. Cronbach’s alpha for the CBI-R groupings (Table 2) demonstrated adequate internal consistency for 8 of the subsections and the total score. Sleep had the lowest Cronbach alpha, while the stereotypic and motor behaviours subsection was just below the recommended alpha value (0.7).

### Validity and utility of the Revised Cambridge Behavioural Inventory (CBI-R)

**Figure 1:**
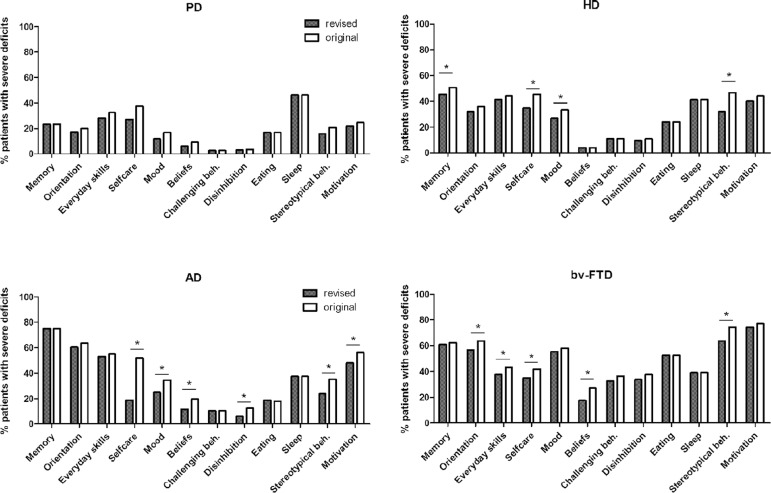
A comparison of the behavioural profiles generated using the original Cambridge Behavioural Inventory (CBI) and the CBI-revised (CBI-R) for Parkinson’s disease (PD), Huntington’s disease (HD), Alzheimer’s disease (AD) and behavioural variant frontotemporal dementia (bv-FTD).

The utility of the CBI-R was tested by identifying behavioural profiles for the four neurodegenerative diseases (Figure 1). Comparison of profiles using ANOVA revealed a significant effect of diagnosis (p<0.05) on all subsections except sleep, corroborating previous findings for the original CBI [10]. Post-hoc analyses revealed overall consistency in the behavioural profiles between the two versions of the CBI for all four patient groups (Figure 1). Using both versions of the CBI, bv-FTD showed the highest prevalence of behavioural symptoms, notably stereotypical behaviours and motivation. Memory and orientation deficits were more common in AD patients, with a prevalence comparable to bv-FTD patients. Severe deficits in ADLs were also common in AD. For PD and HD patients, deficits were similar in most sections, but HD patients had significantly more impairment in memory, mood, motivation and stereotypical behaviour than PD patients. In contrast with the original CBI, the revised version did not show differences between PD and AD in terms of prevalence of mood impairment.

The correlation between the different questionnaire subsections was generally very high (r>0.6, p<0.0001).

## Discussion

We were able to derive a revised, shorter version of the CBI that remains effective in discriminating between behavioural profiles of the different neurodegenerative diseases. The CBI-R has been reduced to 45 items (81 previously), minimising administration time and making it more user friendly. We also made formatting changes to facilitate comprehension of the instructions and questions. The confirmation that items from the revised questionnaire loaded onto virtually identical factors to the original CBI in the PCA analysis suggests a stable solution.^16^ Preliminary analyses demonstrated that the internal consistency remained high in the new questionnaire; although the ‘sleep’ subsection was below adequate levels. ‘Sleep’ is nevertheless clinically highly relevant and hence merits inclusion in the CBI-R.

PCA of the original and revised CBI revealed remarkably good separation of the various putative cognitive, self-care, psychiatric and behavioural domains suggesting that the instrument has ecological and clinical validity, but also that the various questions do, indeed, tap separable aspects of brain dysfunction. This is confirmed by examination of the behavioural profiles observed across the different neurodegenerative disorders which show progressive dysfunction of separable neural systems. Patients with bv-FTD show the highest prevalence of behavioural symptoms, with high rates of apathy, stereotypic behaviours, disinhibition, and abnormal eating.^4^ Recent brain imaging studies using voxel based morphometry have related these symptoms to involvement of the ventral and medial frontal cortex.^18,19^ In AD, deficits in memory, orientation, everyday skills and self-care are dominant, reflecting medial temporal and posterior association cortical pathology. Apathy is also common and may reflect anterior cingulate involvement, perhaps as an indirect consequence of severe posterior cingulate pathology demonstrated by FDG-PET at the very early stage of the disease.^20^ In PD, the overall rate of symptom endorsement by caregivers is low, although the high rate of sleep dysfunction deserves further exploration.^6^ Seriously disruptive and challenging behaviour had a surprisingly low prevalence in HD except for stereotypic patterns of behaviour and poor motivation, which may reflect selective disruption of fronto-striatal circuits as a result of basal ganglia pathology.^21,22^

Our study has some clear limitations. Further validation of the CBI-R against other instruments and the examination of inter-rater and test-retest reliability is desirable.^23^ Validation should also be carried out in an independent cohort of patients. It would also be useful to extend the range of disorders to include vascular dementia, DLB and parkinsonian plus cases.

It is imperative that instruments are available for accurate detection and quantification of behavioural and neuropsychiatric disturbances, as these represent primary manifestations of dementia and cognitive dysfunction.^24^ The CBI and the CBI-R have the advantage of evaluating a wider range of psychopathology than most existing instruments including patients with cognitive deficits but not dementia.^10^ The CBI is also able to elicit information that may distinguish between at least four different neurodegenerative diseases, without requiring specialist training and is filled in by the carer outside the clinic consultation. These properties make these instruments attractive options for use in international clinical and research settings, as well as future drug trials, where they could be employed as a measurement of treatment efficacy.
